# Analysis of Haematological Parameters as Predictors of Malaria Infection Using a Logistic Regression Model: A Case Study of a Hospital in the Ashanti Region of Ghana

**DOI:** 10.1155/2019/1486370

**Published:** 2019-05-21

**Authors:** Ellis Kobina Paintsil, Akoto Yaw Omari-Sasu, Matthew Glover Addo, Maxwell Akwasi Boateng

**Affiliations:** ^1^Kumasi Centre for Collaborative Research in Tropical Medicine, KNUST, Kumasi, Ghana; ^2^Department of Mathematics, Kwame Nkrumah University of Science and Technology, Kumasi, Ghana; ^3^Department of Theoretical and Applied Biology, Kwame Nkrumah University of Science and Technology, Kumasi, Ghana; ^4^Faculty of Engineering, Ghana Technology University College, Ghana

## Abstract

Malaria is the leading cause of morbidity in Ghana representing 40-60% of outpatient hospital attendance with about 10% ending up on admission. Microscopic examination of peripheral blood film remains the most preferred and reliable method for malaria diagnosis worldwide. But the level of skills required for microscopic examination of peripheral blood film is often lacking in Ghana. This study looked at determining the extent to which haematological parameters and demographic characteristics of patients could be used to predict malaria infection using logistic regression. The overall prevalence of malaria in the study area was determined to be 25.96%; nonetheless, 45.30% of children between the ages of 5 and 14 tested positive. The binary logistic model developed for this study identified age, haemoglobin, platelet, and lymphocyte as the most significant predictors. The sensitivity and specificity of the model were 77.4% and 75.7%, respectively, with a PPV and NPV of 52.72% and 90.51%, respectively. Similar to RDT this logistic model when used will reduce the waiting time and improve the diagnosis of malaria.

## 1. Introduction

Apart from loss of valuable life, malaria is the leading cause of morbidity in Ghana representing 40-60% of outpatient hospital attendance with about 10% ending up on admission [1]. In the year 2002, it cost the government of Ghana US$ 50.05 million to control malaria; private businesses in Ghana also lost US$ 6.58 million in 2014 alone as a result of malaria [1, 2]. The burden of malarial infections cannot be underestimated; it is widely agreed that malaria is a disease of the poor [3]. Therefore, it is not surprising that malaria is endemic in Africa where poverty is rampant [3].

Some clinicians think missing a case of malaria is a bigger problem than it is in reality and therefore treat most people with fever for malaria [4]. Overdiagnosis of malaria leads to wastage in the healthcare system; individual may have to pay more, spend more days at the hospital, and also miss work [5]. Due to the socioeconomic implications of misdiagnosis of malaria, it is imperative that investment is made in the accurate diagnosis and management of this disease to improve healthcare outcomes and reduce poverty [5].

In malaria diagnosis, RDTs are relatively simple and quick way of detecting the presence of the parasites in human blood. However, RDTs are expensive to use compared to malaria microscopy and may not be able to detect some infections with low parasitaemia [6]. Another weakness of RDT in the diagnosis of malaria is that it remains positive for about 15 days after successful treatment of the disease [7]. Microscopic examination of peripheral blood film remains the most preferred and the gold standard for malaria diagnosis worldwide [8]. Nonetheless, the specificity, sensitivity, and reliability of this method depend on the procedure for blood collection, processing, and experience of the laboratory technician [8]. Unlike developed countries, the level of skills required for microscopic examination of peripheral blood film is often lacking in Ghana and many other African countries, particularly in rural areas, where most malaria cases occur [8]. The situation is compounded with lack of or use of fake reagents, high workload, unreliable electricity supply, and faulty microscopes [9]. This leads to inaccurate diagnosis of malaria. For example, in Sudan, about 75% of microscopy malaria diagnosis was later found to be false positive [10]. In addition, when diagnosis is based on only signs and symptoms in over 90% of the cases, the disease was misdiagnosed [4].

Though expensive, most medical laboratory scientists and technicians in district hospitals in Ghana have access to haematological analysers; unlike malaria microscopy, they can easily and accurately perform full blood count to assess haematological parameters. Since haematological parameters are known to change with malaria infections, it is necessary to better understand this relationship to improve the diagnosis of this killer disease. This study therefore aims at determining the extent to which haematological parameters and demographic characteristics of patients can be used to predict malaria infection using logistic regression.

## 2. Methods

### 2.1. Data Source

The data for this study was obtained as secondary data from the laboratory department of St. Patrick's Hospital, Offinso, from January 2018 to June 2018 by reviewing the haematological records book. Full blood count and malaria test data from a total of 2076 patients with suspicion of malaria were used, comprising 1200 females and 876 males between the ages of 1 to 102 years. The diagnosis of malaria was done using Rapid Diagnostic Test (RDT) and confirmed using Giemsa staining technique by trained microscopist. Full blood count analyses were done using Mindray BC – 3000Plus auto analyser after careful calibration according to the manufacturer's standards. Haematological parameters used in this study were haemoglobin (Hb), white blood cell (WBC), platelet (Plt), lymphocyte (Lymph), mixed cell count (MXD), and neutrophil (Neut).

For this study, the data obtained was coded as follows: malaria (positive = 1 and negative = 0); gender (1 = male, 0 = female). Age and all haematological parameters used in this study were numeric variables and therefore did not need coding. This study adopts a logistic regression model to analyse haematological parameters as predictors for malaria infection.

### 2.2. Data Analysis

The data obtained was initially entered in Microsoft Excel (2016) and checked for errors after which it was exported to IBM SPSS Statistics 22 software for logistic regression analysis.

### 2.3. Basic Concepts of Logistic Regression

#### 2.3.1. Odds

When the probability of an event of interest occurring is p, then 1-p is probability of that event not occurring. The odds of that event are the ratio between the two probabilities: the probability that an event of interest will occur and the probability that it will not occur.

(1) $$\begin{array}{c} Odds\;\;of\;\;an\;\;event=\frac{p}{1-p} \end{array}$$

In logistic regression the impact of the predictor variables is usually explained in terms of odds, because the odds calculate the probability of the event of interest occurring over the probability of that event not occurring. By using the equation* y *=*α*+*βx, *the mean of the dependent variable y in terms of an independent variable* x *is modelled in linear regression. However, this model is not good enough, because extreme values of* x *will give values of *α*+*βx *that is not within 0 and 1. The logistic regression solves this problem by transforming the odds using the natural logarithm. So, in logistic regression we replace the odds with its natural log (logit) and model it as a linear function of the explanatory variable to obtain the simple logistic model.

(2) $$\begin{array}{c} logit\left(\;y\;\right)=In\left(\;odds\;\right)=In\left(\;\frac{p}{1-p}\;\right)=\alpha +\beta x \end{array}$$

We derive an equation to predict the probability that an event of interest will occur, such as if a patient tests positive for malaria, by taking the antilog of (2) on both sides.

(3) p1−p=eα+βxp=eα+βx1−pp=eα+βx−eα+βx∗pp+eα+βx∗p=eα+βxp1+eα+βx=eα+βxp^=eα+βx1+eα+βxp^=11+e−α+βx

where $\hat{p}$ is the probability statement of the estimated regression equation.

The simple logistic model could be extended to include multiple independent variables; in that case the complex logistic regression model will be constructed as

(4) logityInodds=Inp1−p=α+β1x1+β2x2+…+βmxm

Therefore the estimated probability that a patient tests positive for malaria given the various predictors is

(5) $$\begin{array}{c} \hat{p}=\frac{1}{1+e^{-\left(\alpha +\beta_{1}x_{1}+\beta_{2}x_{2}+\ldots +\beta_{m}x_{m}\right)}} \end{array}$$

#### 2.3.2. Odds Ratio

The odds ratio (OR) compares the odds of two different events. For two different events z and x, the corresponding odds of z occurring relative to x occurring are called the odd ratio.

(6) $$\begin{array}{c} Odd\;\;ratio=\frac{odds\left\{z\right\}}{odds\left\{x\right\}} \end{array}$$

The odds ratio can be calculated from a logistic regression equation by taking the exponential of both sides; when this happens the equation can be rewritten as

(7) $$\begin{array}{c} odds=\left(\;\frac{p}{1-p}\;\right)=e^{\alpha }\times e^{\beta_{1}x_{1}}\times e^{\beta_{2}x_{2}}\times \ldots .\times e^{\beta_{m}x_{m}} \end{array}$$

When all other factors remained unchanged and a variable x_i_ increases by 1 unit, then the odds will increase by a factor *e*^*βi*^. This factor (*e*^*βi*^) gives the relative amount by which the odds of the outcome increase or decrease when the value of the independent variable (x_i_) is increased by 1 unit; therefore, *e*^*βi*^ is the odds ratio (OR) for the independent variable x_i._

## 3. Results

### 3.1. Descriptive Statistics

**Figure 1:**
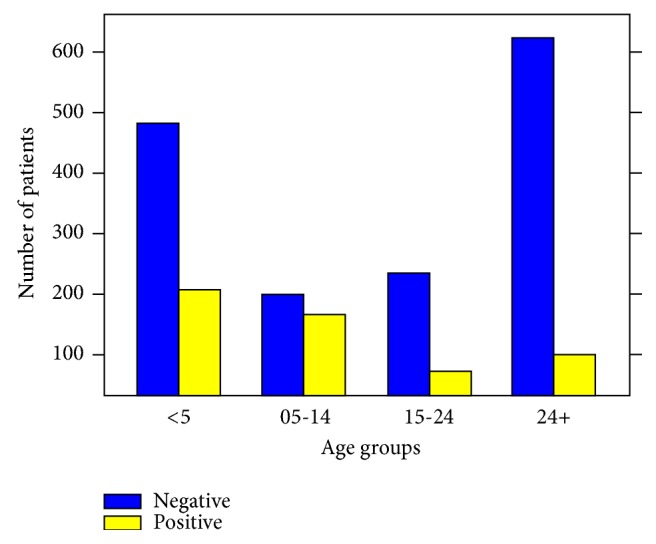
A chart of malaria infection in the various age groups.

Out of the total 2076 patients tested for malaria, 539 (25.96%) were positive. Within the various age groups, children between the ages of 5 and 14 years recorded the highest malaria prevalence of 45.30% (164 out of 362). A total of 206 (29.99%) out of 687 children less than 5 years old tested positive for the disease; adults > 24 years recorded a prevalence of 13.79% (100 out of 725) (Figure 1).

### 3.2. The Logistic Regression Model

A backward stepwise selection of predictors into the binary logistic model produced series of models. Tables 1 and 2 show Wald's test of significance and odds ratio of the predictor variables in the initial and final models, respectively. The first column is the haematological parameters used as predictor variables. The second and third columns are the values of the unstandardized regression coefficients and their standard errors, respectively, while the fourth, fifth, and sixth columns are the Wald test, degree of freedom, and the significance level of the Wald test, respectively. Column seven is the odds ratio and column eight is the confidence interval of the odds ratio.

The final model considering their respective parameters (*β*) for each predictor variable in Table 2 could be written as

(8) logityInodds=Inp1−p=5.164−0.41Age−0.183Hb−0.011Plt−0.031Lymph

This implies that, to predict whether a patient is having malaria or not, the age, haemoglobin (Hb), platelet (Plt), and lymphocyte (Lymph) levels of that patient could be relevant factors. Therefore, a rise in these variables could decrease the probability of a patient getting malaria.

### 3.3. Prediction

Using the patient with laboratory ID number ML052 who is 1 year old with Hb= 8.50g/dL, Plt=73×10^9^/L, and Lymph = 33.40%, the probability that the patient will be classified as having malaria could be calculated by

(9) logityInodds=5.164−0.411−0.1838.5−0.01173−0.03133.40logityInodds=5.164−0.41−1.556−0.803−1.0354logityInodds=1.3596

Hence, the resulting odds ratio (OR) is *e*^1.3596^=3.89, which implies that the probability for the patient with laboratory ID number ML052 to be classified as having malaria is almost four times.

### 3.4. Classification Table with Only the Intercept

Table 3 displays the baseline classification for the model with only the intercept with a predictive ability of 26%. Hence, if one simple guess is made that all patients are diagnosed with malaria infection, one will be able to classify 26% of the patients correctly by chance. However, when predictors are included in the model it is expected that the classification ability will improve.

### 3.5. Classification Test for Final Model

Table 4 displays the percentage of classification for the final model. This classification test shows how well the model is able to identify the correct category, malaria and without malaria, using sensitivity and specificity analysis.

From Tables 3 and 4, the classification percentage accuracy for the initial and final models is 26% and 76.1%, respectively. This shows a significant improvement in the classification of malaria infection when the final model is used. The sensitivity and specificity of the final model can be determined from the classification table (Table 4). From the final model, the sensitivity of model in classifying patients with malaria is (417/(417 + 122)) 77.4% and the specificity of the final model to classify patients without malaria is (1163/(374 + 1163)) 75.7%.

Hence, the positive predictive value = 417/(417 + 374) = 52.72% and the negative predictive value = 1163/(122 + 1163) = 90.51%. This means that the final model is able to predict 52.72% of patients diagnosed with malaria and 90.51% of patients without malaria. A summary of various evaluation tests done on the model to ascertain its diagnostic capabilities is shown in Table 5.

### 3.6. Receiver Operating Characteristics (ROC) Curve Analysis

**Figure 2:**
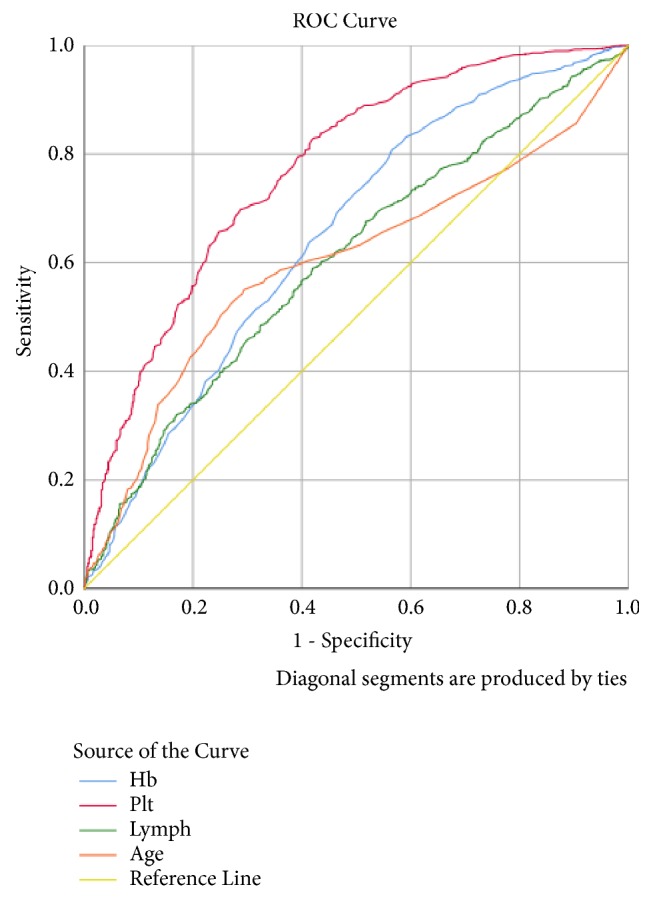
ROC curve of the significant predictors in the final logistic model.

From Figure 2, platelet recorded the highest (0.772) area under the curve. Hb, Lymph, and age recorded ROC values of 0.650, 0.605, and 0.600, respectively. The model has a good predictive ability, since all the significant predictors had ROC values >0.5.

## 4. Discussion

This study identified that 29.99% of children less than five years tested positive for malaria. This is slightly higher than results from a recent nationwide survey where malaria parasite prevalence among children aged 6-59 months was determined to be 27.5% and 20.4% using RDT and microscopy, respectively [11]. This study recorded the highest malaria prevalence of 43.30% among children between the ages of 5 and 14 years. This is consistent with a study conducted in Kenya where highest prevalence of malaria was noted among children aged 11–14 years; another related study observed that children of schoolgoing age (5-18) have the highest risk of getting malaria [12, 13]. It is important for malaria control programmes to recognize that certain age groups such as children between the ages of 5 and 14 years have a higher risk of getting the disease and develop appropriate interventions to address it. Moreover, this study determined the overall prevalence of malaria to be 25.96%; this is lower than the 31.0 % recorded as malaria microscopy test positive rate for the year 2015, in Ghana, but similar to the 26.4% recorded in 2016 [11]. On the contrary, the current finding of 25.96% is higher than reports from other parts of Africa, such as Tanzania (13%) and Northwest Ethiopia (18.4%) [14, 15].

The main objective of this study is to predict malaria infection using gender, age, and haematological parameters. To achieve this goal, a logistic regression model was employed. The first regression step included these parameters: gender, age, haemoglobin, white blood cell, platelet, lymphocyte, mixed cell count, and neutrophil. However, after using the stepwise selection method to select predictors into the binary logistic model, only age, haemoglobin, platelet, and lymphocyte were significant with significance values less than 0.01 as shown in Table 1. A recent study also identified these variables as significant, though, in that study gender and nationality also played a significant role in predicting malaria [16].

From Table 2 which gives the final model in this study, the odds ratios of the significant variables, age, haemoglobin, platelet, and lymphocyte, were 0.96, 0.83, 0.99, and 0.97, respectively. This means that as these variables increase by 1 unit, the odds of getting malaria also decrease by 4%, 17%, 1%, and 3%, respectively. This supports the work of Kleinschmdt and Sharp [17] who reported that malaria is most common in children. Again, it has been reported that low haemoglobin and platelet levels have also been associated with the disease [18, 19]. Furthermore, the Wald statistics for these variables were significantly different from zero; thus, they substantially contribute towards predicting the outcome of the model.

In this study, the sensitivity and specificity of the logistic model to predict malaria infection were 77.4% and 75.7%, respectively. The current model with sensitivity of 77.4% is slightly higher than RDT (62.5%) and similar to presumptive diagnosis (70.83%) of malaria in Ho, Ghana [20]. Nonetheless, a specificity of 75.7% observed in the current model is lower than RDT (92.73%) but significantly higher than presumptive diagnosis (25.5%) [20]. This shows that this model is far better in predicting malaria than diagnosis only based on clinical signs and symptoms.

The PPV and NPV of the final model developed were 52.72% and 90.51%, respectively. Even though this model has a high sensitivity of 77.4%, only 52.72% of the positive cases truly have malaria. However, the model is very effective in identifying malaria negative cases since 90.51% of all the malaria negative cases identified by this model truly do not have the disease. A malarial study conducted in Kenya obtained high negative predictive values comparable to the current model for most haematological parameters investigated but recorded slightly higher PPV for platelet <150 x10^3^/*μ*L (69%), Hb <10g/dL (60%), and significantly lower for lymphocytes counts <2000/*μ*L (6%) [21]. Furthermore, the PPV value of 52.72% for the current model is comparable to the 56.8% observed for PCR for malaria diagnosis, but lower than 67.7% and 66.9% recorded for RDT and microscopy, respectively [22]. The NPV for the final model developed from this study was 90.51%, which is higher than studies done in Nigeria to find the NPV of RDT (70.73%), microscopy (75.0%), and PCR (84.5%) in diagnosing malaria [22, 23]. But the current finding is similar to the overall NPV of 94.2% noted for RDTs used for diagnosis of malaria among under five children [24]. This shows that the diagnostic ability of the present model is comparable to other diagnostic tools used to diagnose malaria.

*Limitations of the Study.* The haematological analyser produced 19 parameters; however, the secondary data contained only the six prominent ones which were used in the model. It is possible that some of the unused parameters, when included in the model, could produce a better model than the current one.

The data used contained more females than males; if females have a different risk of malaria infection than men, then it will affect the overall estimates of malaria indices in the catchment communities and this may lead to wrong interpretation and policy initiatives.

Approximately half of the patients sampled were children aged not more than 14 years and since children have more malaria cases than adults, this might affect the overall reported diseases prevalence in the population.

## 5. Conclusion

The binary logistic model developed for this study identified age, haemoglobin, platelet, and lymphocyte as the most significant predictors. The overall prevalence of malaria in the study area was determined to be 25.96%. The final logistic model obtained for use in the diagnosis of malaria is In(odds) = In(p/(1 − p)) = 5.164 − 0.41Age − 0.183Hb − 0.011Plt − 0.031Lymph.

The sensitivity and specificity of the above model were 77.4% and 75.7%, respectively, with a PPV and NPV of 52.72% and 90.51%, respectively. Similar to RDT this logistic model when used will reduce the waiting time and improve the diagnosis of malaria.

## Figures and Tables

**Table 1:** Wald's test of significance and odds ratio of predictor variables in the initial model.

Variable	*β*	S.E (*β*)	Wald	df	Sig.	e^*β*^(OR)	95% CI for OR	
							lower	upper
Age	-0.041	0.004	111.381	1	<0.01	0.960	0.953	0.967
Gender	-0.040	0.125	0.102	1	0.749	0.961	0.752	1.227
Hb	-0.187	0.028	42.974	1	<0.01	0.830	0.785	0.877
WBC	-0.009	0.013	0.489	1	0.484	0.991	0.966	1.016
Plt	-0.011	0.001	252.678	1	<0.01	0.989	0.988	0.990
Lymph	-0.032	0.004	50.767	1	<0.01	0.969	0.961	0.977
Mxd	0.000	0.021	0.000	1	0.985	1.000	0.960	1.042
Neut	0.000	0.02	0.000	1	0.985	1.000	0.960	1.042
Constant	5.317	0.455	136.832	1	<0.01	203.824		

Hb = Haemoglobin (g/dL); Wbc = White blood cell(×10^9^/L); Plt = Platelet (×10^9^/L); Lymph = Lymphocyte (%); Mxd = Mixed Cell Count (%); Neut = Neutrophil (%).

**Table 2:** Wald's test of significance and odds ratio of predictor variables in the final model.

Variable	*β*	S.E (*β*)	Wald	df	Sig.	e^*β*^(OR)	95% CI for OR	
							lower	upper
Age	-0.041	0.004	117.143	1	<0.01	0.960	0.953	0.967
Hb	-0.183	0.028	42.764	1	<0.01	0.833	0.788	0.880
Plt	-0.011	0.001	258.940	1	<0.01	0.989	0.988	0.990
Lymph	-0.031	0.004	54.204	1	<0.01	0.969	0.961	0.977
Constant	5.164	0.370	194.749	1	<0.01	174.915		

Hb = Haemoglobin (g/dL), Plt = Platelet (×10^9^/L), Lymph = Lymphocyte (%),

**Table 3:** Classification table with only the intercept.

Observed	Predicted		
	With malaria (1)	Without malaria (0)	Percentage correct
With malaria (1)	539	0	100
Without malaria (0)	1537	0	0
Overall percentage			26

**Table 4:** Classification table for the final model.

Observed	Predicted		
	With malaria (1)	Without malaria (0)	Percentage correct
With malaria (1)	417	122	77.4
Without malaria (0)	374	1163	75.7
Overall percentage			76.1

**Table 5:** Summary of various tests conducted to evaluate the model.

Model Evaluation Test Statistic	Result	95% CI
Overall Disease Prevalence	25.96%	24.09% - 27.91%

Sensitivity	77.37%	73.60% - 80.83%

Specificity	75.67%	73.44% – 77.79%

Positive Predictive Value	52.72%	50.24% - 55.18%

Negative Predictive Value	90.51%	89.05% - 91.78%

Positive Likelihood Ratio	3.18	2.88 – 3.51

Negative Likelihood Ratio	0.30	0.26 – 0.35

Model Accuracy	76.11%	74.21% – 77.93%

## Data Availability

The data used to support the findings of this study are available from the corresponding author upon request.
